# Management of Treatment-Resistant Bipolar Disorder with Psychotic Features and The Adverse Reactions of Antipscyhotics

**DOI:** 10.1192/j.eurpsy.2026.11204

**Published:** 2026-07-31

**Authors:** D. Karaçam, M. M. Kırpınar, Ö. F. Demirel

**Affiliations:** Department of Psychiatry, Istanbul University Cerrahpasa Faculty of Medicine, Istanbul, Türkiye

## Abstract

**Introduction:**

Treatment-resistant bipolar disorder with psychotic features is a major challenge in psychiatry. It is associated with relapse, aggression, and suicidal behaviour. Although indicated for schizophrenia, clozapine benefits refractory bipolar disorder, reducing aggression and suicidality. However, its use is limited by adverse reactions, including seizures and endocrine disturbances.

**Objectives:**

To present a case of treatment-resistant bipolar disorder with psychotic features, focusing on the management of clozapine-related seizures and paliperidone-induced hyperprolactinaemia.

**Methods:**

Clinical information was obtained from the patient’s psychiatric and neurological assessments during hospitalization, documenting treatment trials, adverse effects, and management strategies.

**Results:**

We report the case of a 27-year-old woman with a 9-year history of bipolar I disorder and two suicide attempts by overdose. She had failed multiple trials of valproate, risperidone, aripiprazole, olanzapine and paliperidone. Her most recent admission was marked by severe manic and psychotic symptoms with minimal response to haloperidol, amisulpride, and chlorpromazine. Electroconvulsive therapy (ECT) was initiated, after a few sessions the improvement was insufficient. Clozapine was then introduced and titrated while ECT continued in parallel. She received 12 ECT sessions before discontinuation. The patient’s psychotic features and aggression had resolved.

Following a 32-day period of hospitalisation, she was discharged with clozapine, lithium, paliperidone and quetiapine. 13 days later, she was readmitted with seizures following myoclonus. She was referred to neurology, an electroencephalogram (EEG) was performed, and primary generalised epilepsy was diagnosed. Lamotrigine was gradually introduced. Given the patient’s history of refractory psychotic mania, the decision was made not to discontinue clozapine, but rather to reduce the dosage from 500 mg/day to 300 mg/day. Following this adjustment, she didn’t experience any further seizures and there was an improvement in her psychotic and mood symptoms.

Subsequently, the patient developed secondary amenorrhoea and hyperprolactinaemia (119 ng/mL), attributed to paliperidone palmitate. Reducing its dosage and adding low-dose aripiprazole (2.5 mg/day) successfully decreased prolactin levels.

**Conclusions:**

This case illustrates the therapeutic dilemma of balancing psychiatric efficacy with neurological safety. Clozapine can improve psychotic and manic symptoms in treatment-resistant bipolar disorder, even after seizures, when dose-adjusted. Consequently, early discontinuation of the medication, carrying high risk of exacerbation, was prevented. Paliperidone-induced hyperprolactinaemia was managed with dose reduction and adjunctive aripiprazole. Individualised approaches are crucial where risk–benefit decisions deviate from guidelines.

**Disclosure of Interest:**

None Declared

